# Engaging Stakeholders in the Design of One Health Surveillance Systems: A Participatory Approach

**DOI:** 10.3389/fvets.2021.646458

**Published:** 2021-05-24

**Authors:** Marion Bordier, Flavie Luce Goutard, Nicolas Antoine-Moussiaux, Phuc Pham-Duc, Renaud Lailler, Aurelie Binot

**Affiliations:** ^1^ASTRE, Univ Montpellier, CIRAD, INRAE, Montpellier, France; ^2^CIRAD, UMR ASTRE, Montpellier, France; ^3^Faculty of Veterinary Medicine, Kasetsart University, Bangkok, Thailand; ^4^CIRAD, UMR ASTRE, Bangkok, Thailand; ^5^Fundamental and Applied Research for Animals and Health Research Unit, Faculty of Veterinary Medicine, University of Liege, Liege, Belgium; ^6^Center for Public Health and Ecosystem Research, Hanoi University of Public Health, Hanoi, Vietnam; ^7^Institute of Environmental Health and Sustainable Development (IEHSD), Hanoi, Vietnam; ^8^Laboratoire de Sécurité des Aliments, Agence Nationale de Sécurité Sanitaire de l'Alimentation, de l'Environnement et du Travail (ANSES), Maisons-Alfort, France; ^9^Université Paris-Est Créteil Val de Marne, Créteil, France

**Keywords:** antimicrobial resistance, co-construction, One Health, participatory, *Salmonella*, surveillance

## Abstract

Many One Health surveillance systems have proven difficult to enforce and sustain, mainly because of the difficulty of implementing and upholding collaborative efforts for surveillance activities across stakeholders with different values, cultures and interests. We hypothesize that only the early engagement of stakeholders in the development of a One Health surveillance system can create an environment conducive to the emergence of collaborative solutions that are acceptable, accepted and therefore implemented in sustainable manner. To this end, we have designed a socio-technical framework to help stakeholders develop a common vision of their desired surveillance system and to forge the innovation pathway toward it. We implemented the framework in two case studies: the surveillance of antimicrobial resistance in Vietnam and that of *Salmonella* in France. The socio-technical framework is a participatory and iterative process that consists of four distinct steps implemented during a workshop series: (i) definition of the problem to be addressed, (ii) co-construction of a common representation of the current system, (iii) co-construction of the desired surveillance system, (iv) identification of changes and actions required to progress from the current situation to the desired situation. In both case studies, the process allowed surveillance stakeholders with different professional cultures and expectations regarding One Health surveillance to gain mutual understanding and to reconcile their different perspectives to design the pathway toward their common vision of a desired surveillance system. While the proposed framework is structured around four essential steps, its application can be tailored to the context. Workshop facilitation and representativeness of participants are key for the success of the process. While our approach lays the foundation for the further implementation of the desired One Health surveillance system, it provides no guarantee that the proposed actions will actually be implemented and bring about the required changes. The engagement of stakeholders in a participatory process must be sustained in order to ensure the implementation of co-constructed solutions and evaluate their effectiveness and impacts.

## Introduction

The One Health concept calls for systemic approaches to better understand and manage complex health problems. This requires the bridging of activities carried out in the human, animal, and environmental health sectors, mobilizing the different professions and decision-making scales, and establishing interdisciplinary approaches that bring together biomedical, environmental, and social sciences (1).

International organizations, governments, and the scientific community are widely promoted the application of the One Health concept to surveillance when it deals with complex health hazards such as zoonotic diseases, antibiotic resistance, or biological and chemical contaminants in the food chain (2, 3). The approach highlights potential improvements of surveillance in terms of epidemiological and economic performance. Ultimately, it is expected to improve knowledge of health events and their management, while reducing the costs associated with surveillance activities and interventions (4–6). However, a wide range of technical, organizational, and sociological factors is impeding the sustainable implementation of One Health surveillance (7–12).

Surveillance mobilizes networks of stakeholders with specific roles and missions subject to their own constraints. It produces information for different categories of beneficiaries with different expectations (13). Although surveillance is most often associated with positive impacts (improvement of the prevention and management of health events), it can have negative repercussions for certain stakeholders (destruction of food products following the detection of health hazards, slaughtering of animals following the detection of certain diseases). This diversity of values, cultures, and interests that coexist within a surveillance system is even more prevalent in a One Health surveillance system where the variety of stakeholders is broader (14). This results in the coexistence of a multiple of representations of the current surveillance system and of changes to improve it, which restrains collective action toward the implementation of One Health surveillance (15, 16). We hypothesize that the collective construction of a common representation of the desired One Health surveillance system is likely to foster mutual understanding among stakeholders and to let emerge collective solutions to operationalize collaboration (17). In addition, the early involvement of stakeholders in collective decision-making should improve their adherence to the proposed solutions and thus their commitment to implementation (18, 19).

To this end, we have developed a socio-technical framework to help stakeholders to construct a common vision of their desired One Health surveillance system and to identify the solutions to make it operational. The framework is an actor-based process, composed of several participatory tools, and implemented during a series of workshops with representatives of surveillance stakeholders. It guides participants in the definition of the causal links between their vision and the changes and actions required to achieve it, so they progressively build the innovation pathway that lays the foundations for the further implementation of the One Health surveillance system. We applied this participatory process to two case studies, the surveillance of antimicrobial resistance (AMR) in Vietnam and the surveillance of *Salmonella* in France. In Vietnam, the government has promulgated a national strategy to combat antimicrobial resistance, including provisions for the establishment of an integrated surveillance system including surveillance activities in the animal health, human health, and environmental sectors (7). Within this context, we offered to support the surveillance stakeholders in defining how the multi-sectoral system would be organized and operate in response to the governmental inquiry. In France, a technical work group, consisting of public and private partners, has been established to optimize the surveillance of *Salmonella* through a better coordination of surveillance activities in the different sectors and at all stages of the food chain (20). We guided the work group in their collective reflection to define their desired surveillance system and the changes needed to establish it.

## Materials and Methods

**Figure 1:**
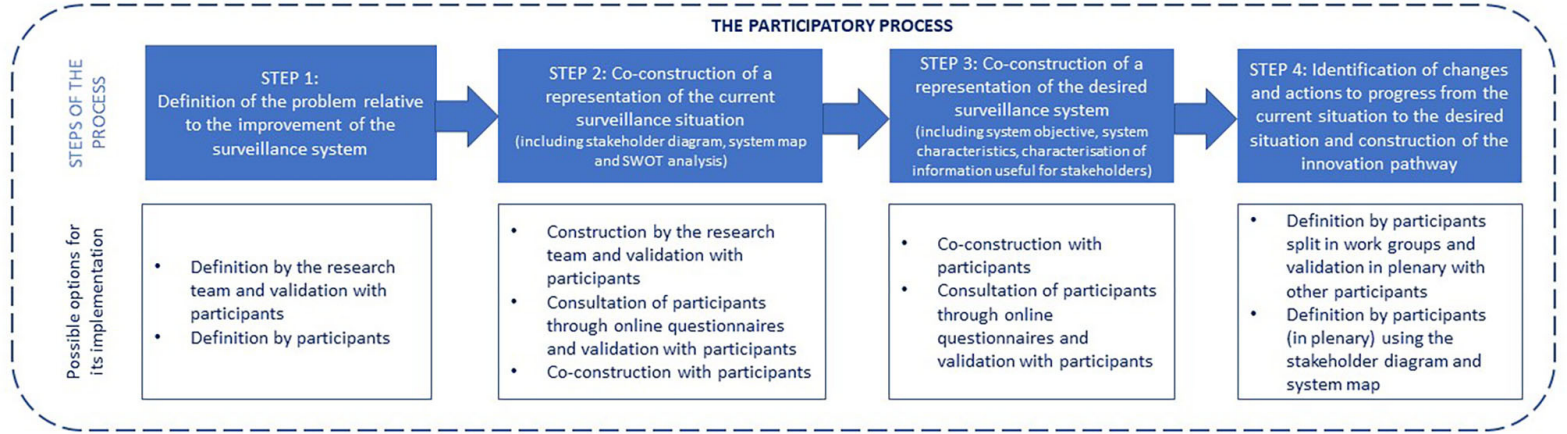
The socio-technical framework structure and its application modalities.

We have developed and applied a socio-technical framework, which is intended to be implemented during a series of workshops. It consists of four steps: (i) definition of the problem to be addressed based on participant expectations, (ii) co-construction of a common representation of the system in place, (iii) co-construction of the desired surveillance system, (iv) identification of changes and actions required to progress from the current situation to the desired situation and construction of the innovation pathway (Figure 1). This framework is implemented using various participatory tools, which can be applied differently depending on the context of implementation and on the information gathered during the process. The description of the case studies illustrates its application to two different epidemiological and socio-political contexts.

Below, we first explain how the workshops were organized and then explain, in detail, the four steps of the framework and how we applied it to the two case studies, using different participatory tools (Table 1).

**Table 1:** Description of the process implementation in Vietnam (surveillance of antimicrobial) and in France (surveillance of *Salmonella*).

	**Steps of the socio-technical framework**			
	**Step 1: Definition of the problem to address**	**Step 2: Co-construction of the representation of the current situation of the surveillance system**	**Step 3: Definition of the desired surveillance system**	**Step 4: Definition of changes and actions required to achieve the desired surveillance system**
Surveillance of AMR in Vietnam	Validation of the problem with participants (plenary discussion) (WS 1)	Building the stakeholder diagram and system map using information shared by participants (plenary discussion using cards) (WS 1) SWOT implementation (plenary discussion) (WS 1)	Definition of the objective of the desired surveillance system and of its core characteristics (plenary discussion) (WS 2)	Identification of changes and actions during plenary discussion (WS 2) and then during group work followed by validation in plenary (WS 3)
Surveillance of *Salmonella* in France	Definition of the problem with participants (plenary discussion using cards) (WS1)	Refining the stakeholder diagram and system map (designed by the research team) with information shared by participants (plenary discussion) (WS 1) Characterization of the desired surveillance system applying thematic analysis to expectations shared by participants (plenary discussion using cards) (WS 1) SWOT implementation (on-line questionnaire)	Characterization of the desired surveillance system using thematic analysis on participants' expectations (WS 1) and characterization of the useful information (group work followed by validation in plenary) (WS 2)	Identification of changes and actions during group work (WS 2 and 3) and followed by validation in plenary (WS 3)

*WS, workshop*.

### Organization of the Workshop Series

The four steps of the socio-technical framework were implemented during three half-day workshops for each case study.

The selection of workshop participants was crucial because their representativeness would determine the richness and relevance of the results produced. As the objective of the process was to gain a fully comprehensive vision of the surveillance systems, it was necessary for all surveillance functions to be represented among participants, while avoiding an over-representation of any one category of stakeholders. In the case of Vietnam, potential participants were identified based on the results of a previous stakeholder analysis study (7). All categories of stakeholders operating in or influencing the system were considered and invited (Table 2). In France, no new recruitment was required as the participants were actually the members of the technical work group (Table 3).

**Table 2:** Description of the stakeholders invited and participating in the participatory process in Vietnam.

**Sector**	**Professional category**	**Invited**	**Participating**		
			**First workshop**	**Second workshop**	**Third workshop**
Multi-sectoral	Authorities (national level)	1	0	1	3
Animal health	Authorities (national level)	3	2	0	0
	National research institutes	1	1	2	2
	International research or technical institutes	1	0	0	1
	International organizations	1	1	4	1
	Pharmaceutical and feed companies	2	1	2	1
Human health	Authorities (national level)	2	1	0	1
	National research institutes	1	0	0	1
	Practitioners (hospitals)	2	0	0	1
	International research or technical institutes	2	3	1	2
	International organizations	1	0	0	1
Food safety	Authorities (national level)	1	1	1	1
	National research institutes	1	0	0	0
Environment	Authorities (national level)	1	0	0	0
	Total	20	10	11	15

**Table 3:** Description of the stakeholders invited and participating in the participatory process in France.

**Sector**	**Professional category**	**Participating**		
		**First workshop**	**Second workshop**	**Third workshop**
Animal health	Scientific or technical institutes	6	2	5
	Professional organizations	4	4	4
Food safety	Authorities	2	2	2
	Scientific or technical institutes	3	3	2
	Professional organizations	7	4	5
Feed safety	Authorities	1	0	0
	Scientific or technical institutes	1	1	1
	Professional organizations	3	2	3
	Total^*^	21	15	18

* *Participants may belong to several categories*.

Before starting discussions, all participants were informed about the organization of the full process.

The workshops were facilitated by pairs of researchers, selected for their ability to lead discussion groups and handle participatory tools and for their legitimacy, in the eyes of the participants, in dealing with the subjects discussed. The choice of facilitators is an important element that influences the success of the participatory process. The facilitators ensured that each participant had the opportunity to express his/her opinion. They encouraged participants to clarify their ideas when too generic or subject to confusion, rewording them when necessary, and obtained general approval from the audience. In Vietnam, facilitation was provided by a researcher who had participated in the development of the methodological framework and had a good knowledge of the system and its stakeholders, and by an academic who is used to facilitating group discussions on cross-cutting health issues. In France, both facilitators had participated in the development of the framework. One had a good knowledge of the system in place; the other had a strong experience in the application of participatory tools and systemic approaches.

For each workshop organized, two observers were designated. Their role was to record the discussions among participants and with the facilitator by taking handwritten notes and pictures.

At the beginning of each workshop, the results of the previous workshop were presented so that participants could reflect on previous work, provide comments, make changes or clarify points, if necessary.

### First Step: Definition of the Problem to Address

**Figure 2:**
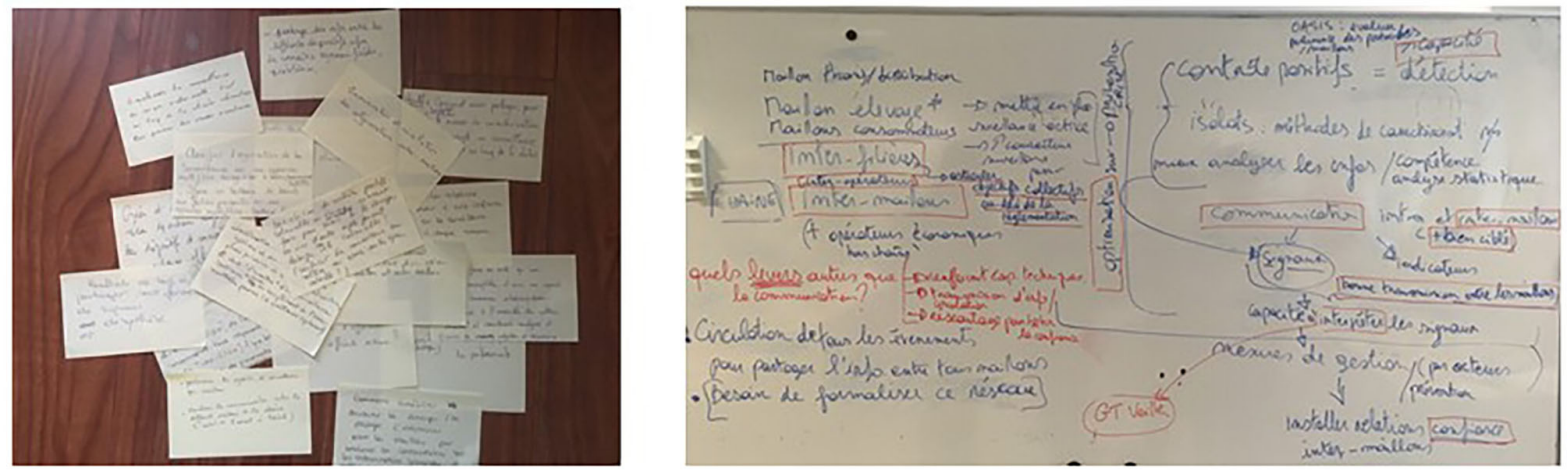
Expectations individually expressed by participants **(left)** and thematic analysis and coding to define the common problem to address **(right)** during the workshops in France.

The reason behind the willingness to implement a One Health surveillance system varies depending on the context and may be perceived differently by participants. In this first step, therefore, we helped participants to express the problem to be addressed in terms of improvement of the current surveillance situation. The objective was to obtain a clear formulation of the problem in terms that everyone could understand and that reflected a common interest for the process. This step was also intended to strengthen participants' commitment to the process by clearly explaining the problem they wished to address through their participation in the workshops. In Vietnam, an inter-ministerial strategy to combat AMR had called for the establishment a multi-sectoral surveillance system and surveillance stakeholders had expressed the need for a multi-stakeholder platform where they could discuss the most appropriate collaborative modalities to implement (7). The issue was therefore predefined but required clarification at the beginning of the first workshop to ensure consensus on the scope of the process and the terminology used. In France, the implementation of the framework was part of the technical group's work plan, but it was necessary to clearly redefine the expectations of each participant engaged in the process in order to collectively formulate a question that obtained full consensus. At the beginning of the first workshop, all participants were asked to write on cards their expectations regarding their involvement in the process. An analysis and thematic codification of expectations were carried out as they were formulated by participants in order to obtain a single, concerted question (Figure 2).

### Second Step: Co-construction of the Representation of the Current Situation of the Surveillance System

The representation of the current situation was determined by describing the current organization and functioning of the surveillance system through three outputs: a diagram of stakeholder interaction within the system (stakeholder diagram), a description of the surveillance programs that are part of the system (system map), and an analysis of the strengths, weaknesses, opportunities, and threats (SWOT) of the system within the context of shift toward One Health surveillance.

#### Stakeholder Diagram

The method used to build the stakeholder diagram was inspired from the PARDI (Problem, Actors, Dynamics, Resources, Interactions) method. It was developed by the ComMod^1^ community to help stakeholders to conceptualize the system surrounding the problem they wish to address and to find solutions to solve the problem (21). It leads to the emergence of a shared representation of the system, integrating the respective knowledge, point of view and expertise of all the participants (22). The process is also an opportunity for participants to learn from each other and to generate new knowledge, allowing for the development of mutual understanding (23).

**Figure 3:**
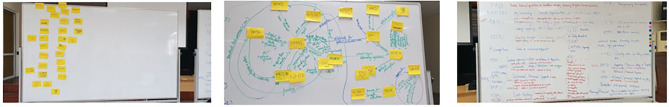
Identification of surveillance stakeholders **(left)** and of interactions between them **(middle)** together with their role and responsibilities **(right)** in the surveillance system of antimicrobial resistance in Vietnam.

**Figure 4:**
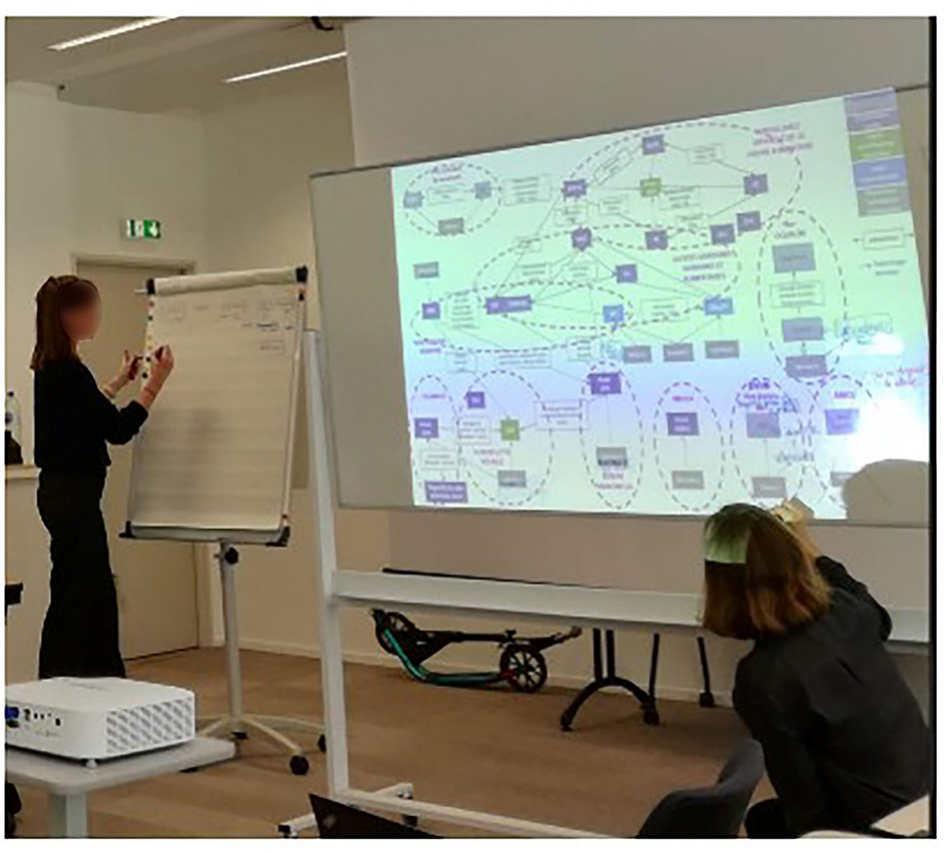
Revision of the stakeholder diagram with the participants of the first workshop in France.

In our framework, we applied the PARDI tool to obtain a stakeholder diagram representing all the stakeholders involved in or impacted by the surveillance system, identifying their roles and missions in relation to surveillance and characterizing the interactions between them. This type of diagram can be developed in different ways. In Vietnam, the entire diagram was co-constructed by combining, in a concerted manner, the information given by the participants during the first collective workshop. Using cards and white boards, facilitators gathered information on main surveillance stakeholders, interactions between them, and their role and responsibilities in the surveillance system (Figure 3). In France, a draft stakeholder diagram focusing on information flows was drawn up by the facilitators on the basis of available information and then submitted for amendment and validation to the participants of the first workshop. The diagram was projected on a white board and participants were invited to bring necessary changes using markers (Figure 4).

#### System Map

Once the stakeholder diagram was complete, stakeholders interacting in the same surveillance program were grouped together to clearly identify the stakeholder network specific to each program. The need to move from an actor-centered to a program-centered representation emerged during the course of the study to highlight collaboration existing between surveillance programs for the governance and/or the implementation of integrated surveillance activities, those collaboration being at the heart of One Health surveillance. For the two case studies, the system map was constructed by the facilitators on the basis of the information collected during the first workshop and then validated during the second workshop with the participants.

#### Identification of the Strengths, Weaknesses, Opportunities, and Threats (SWOT) for the Current Surveillance System

Participants were then asked to conduct a SWOT analysis, i.e., to identify the strengths (S) and weaknesses (W), both internal to the current system as well as existing external threats (T) and unexploited opportunities (O) relative to a shift toward a more effective system (24). Weaknesses and threats are, respectively, the internal and external obstacles that must be addressed to improve the surveillance system; strengths and opportunities are elements that can be used to remove these obstacles. In this participatory process, the SWOT analysis is used as snapshot of the current situation to trigger participant reflection on the need for surveillance improvement. In Vietnam, this work was conducted at the end of the first workshop, by asking participants to propose strengths, weaknesses, opportunities, and threats in turn. The thematic coding progress was done *a posteriori* by the research team. In France, this work was carried out through a questionnaire sent to the participants, with the grouping of results presented at the second workshop.

### Third and Fourth Steps: Definition of the Desired Surveillance System and Necessary Changes

Once participants had agreed on a common representation of the surveillance system, the next step was for them to define their desired surveillance system and build the pathway to reach it. During these two stages, the methodology used in the participatory process referred to the ImpresS method developed by Cirad (the French Agricultural Research Center for International Development) to better consider the impact when constructing a research intervention. It is a participatory, iterative and adaptive process enabling stakeholders to formulate a common vision based on the desired and most convincing impact pathway that the innovation process should follow (25, 26). The impact pathway is a tool grounded in the theory-driven evaluation literature (27). It represents and makes explicit the causal links between the inputs (resources used by the research team), the outputs of the research activities (knowledge, training, technology, etc.), outcomes (e.g., appropriation of the outputs by people), and impacts. We mobilized this framework to define the causal links between actions and changes proposed by participants and their vision of the desired surveillance system (characteristics and objective).

#### Definition of the Desired Surveillance System

In Vietnam, the approach was to lead the workshop participants to define a concerted objective for an optimal multi-sectoral AMR surveillance system. To this end, an open discussion with the whole group was initiated to encourage participants to develop their views on the most relevant objective and purpose for the system in the mid-term (3–5 years). Their different proposals were discussed with the aim of agreeing upon a common objective, reflecting the views of the different participants. On this basis, the system characteristics required to meet this objective were identified.

In France, the desired system was first defined according to the expectations expressed by participants during the first workshop during which they defined the problem to address. As the latter focused on the circulation of useful information, in a second step, the participants further characterized the information they deemed useful for their activities. To this end, the participants were divided into three homogeneous groups according to their main professional category (competent authorities, research and technical institutes, professional organizations) and were asked to identify up to five types of information that they considered useful for *Salmonella* risk management within the context of their mission. They then qualified the information according to type, format required, existence/location, accessibility, use, and valorization.

#### Definition of Changes and Actions Required to Achieve the Desired Surveillance System

During this last step, participants reflected on all the information produced in the previous steps to identify changes and actions required for the operationalization of their vision of the desired surveillance system. By articulating these changes and actions with the representation of the desired surveillance system, we obtained a graphical representation of the stakeholders' theory of change.

In Vietnam, participants were first questioned, in the light of the SWOT analysis results, about the changes to be brought to the current surveillance system to meet the previously defined objectives and characteristics. The changes could target a reorganization of surveillance activities in terms of governance and implementation (addition or removal of a stakeholder; revision, addition or removal of an interaction or action), changes in stakeholder posture, capacity and resources, or any other type of changes relevant to them. Changes proposed by participants, once validated by the entire audience, were directly reported on the stakeholder diagram co-constructed in the second step that was projected on a white board. Then, participants were divided into two homogeneous groups, one consisting of people working in the human health sector and the other of people working in the animal health and food safety sectors. They were asked to rank identified changes according to priority and to propose concrete actions to implement the most important. The results of each group were then presented, discussed, and amended by the other participants.

In France, participants were asked to identify the changes they considered necessary to ensure that useful information could flow properly. To do this, participants split up into groups of three to four people and brainstormed on three changes to be implemented as a priority to promote the flow of useful information. To feed their reflections, they referred to the outputs produced in the previous steps (representation of the desired system, mapping of useful information, SWOT results). The proposed changes could be general -relative to the system—or concern a specific stakeholder. They could be of different types: changes in practices (e.g., actions that stakeholders should do differently), changes in knowledge/capacity (e.g., type of knowledge or capacity the stakeholders should acquire), changes in posture (e.g., type of perception and motivation required by the stakeholders), changes in interaction (e.g., type of interactions the stakeholders should develop). The changes identified were then shared and a thematic analysis was carried out with the whole group to identify, in a concerted manner, the major changes to be implemented in order to reach the desired system.

Figure 1 summarizes possible modalities to apply the socio-technical framework in the different steps.

## Results

### Surveillance of AMR in Vietnam

In Vietnam, the participatory process was implemented during three half-day workshops between December 2018 and January 2019. The participants were from the human health, animal health and food safety sectors. Their number varied between workshops as described in Table 2. For the majority of institutions, only one representative attended the workshops. Two institutions withdrew from the process after the first workshop because they considered that their activity was not directly related to AMR surveillance (environmental authorities) or because they had delegated their surveillance mission to a third party (animal health authorities).

#### Definition of the Problem

During the first workshop, participants agreed on the boundaries of the AMR surveillance system that would be the subject of their discussion. They decided to concentrate on resistance to antibiotics only, while the organization and functioning of the surveillance of antibiotic use would not be addressed. In addition, research and epidemiological surveys would not be considered as surveillance programs unless repeated over time.

#### Representation of the Current Surveillance System

##### Stakeholder Diagram and System Map

**Figure 5:**
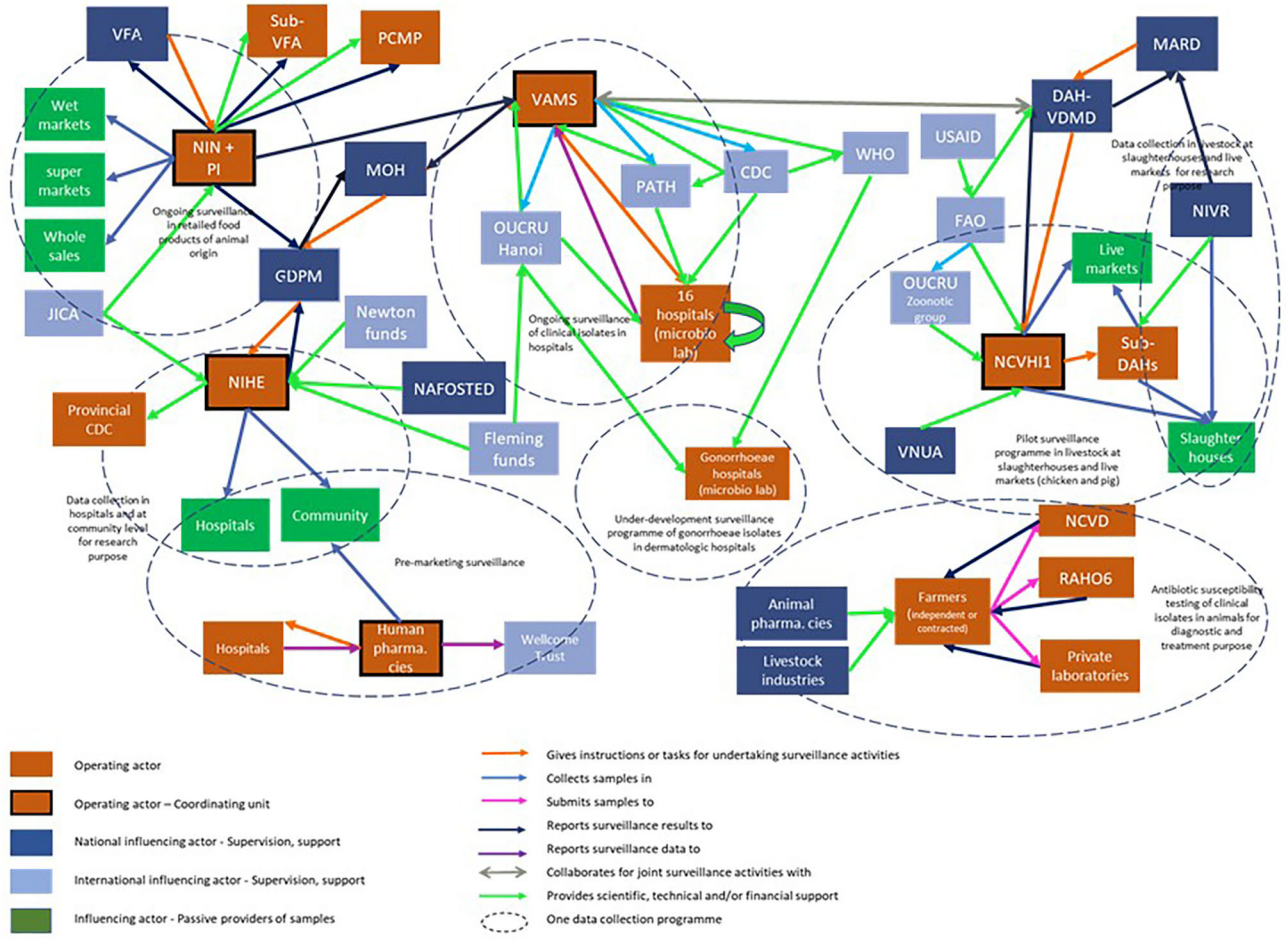
Stakeholder diagram for the surveillance system of antimicrobial resistance in Vietnam. CDC, center for disease control; DAH, department of animal health; FAO, Food and agriculture organization; GDPM, General department of preventive medicine; JICA, Japanese international cooperation agency; MARD, Ministry of agriculture and rural development; MOH, Ministry of Health; NAFOSTED, National Foundation for Science and Technology Development; NCVD, National center for veterinary diseases; NIHE, National institute of hygiene and disease control and prevention; VAMS, Vietnam administration of medical services; VFA, Vietnam food administration; VNUA, Vietnam National University of Agriculture; NCVHI1, National center for veterinary hygiene 1; NIN, National institute of nutrition; NIVR, National institute of veterinary research; WHO, World health organization; OUCRU, Oxford University Clinical Research Unit; PATH-CDC, PATH program of the United States Center for disease control and prevention; PI, Pasteur institutes; RAHO6, Regional animal health organization 6; USAID, United States agency for international development; US-CDC, United Stator Center for disease control.

The stakeholder diagram was developed collectively during the first workshop and revised at the beginning of the second workshop (Figure 5).

In Vietnam, the authorities have initiated three surveillance programs: clinical isolates in hospitals, commensal and zoonotic bacteria in animal commodities, and commensal and zoonotic bacteria in healthy animals. The most accomplished surveillance system is that of human clinical isolates, which is deployed in a network of 16 central and regional hospitals and has long received technical and financial support from foreign research institutes. Surveillance in food or in animals is managed by a lead institution—either a national research institution or a public laboratory—which carries out most of the tasks (coordination, collection and laboratory analysis, data analysis and interpretation, scientific and technical support). Conversely, surveillance in hospitals is much less centralized and involves a wide variety of stakeholders. The local authorities are not involved in any surveillance networks other than for retail food. The authorities in charge of the surveillance programs in the different domains—food-producing animals, retail food, and hospital patients—operate in silos with a lack of coordination. Governmental institutions involved in AMR surveillance are also poorly connected within the same sector.

Simultaneously, the pharmaceutical industry conducts pre-marketing resistance surveillance programs for antibiotics in hospitals and among the population. The organization of these surveillance programs varies from one area to another.

**Figure 6:**
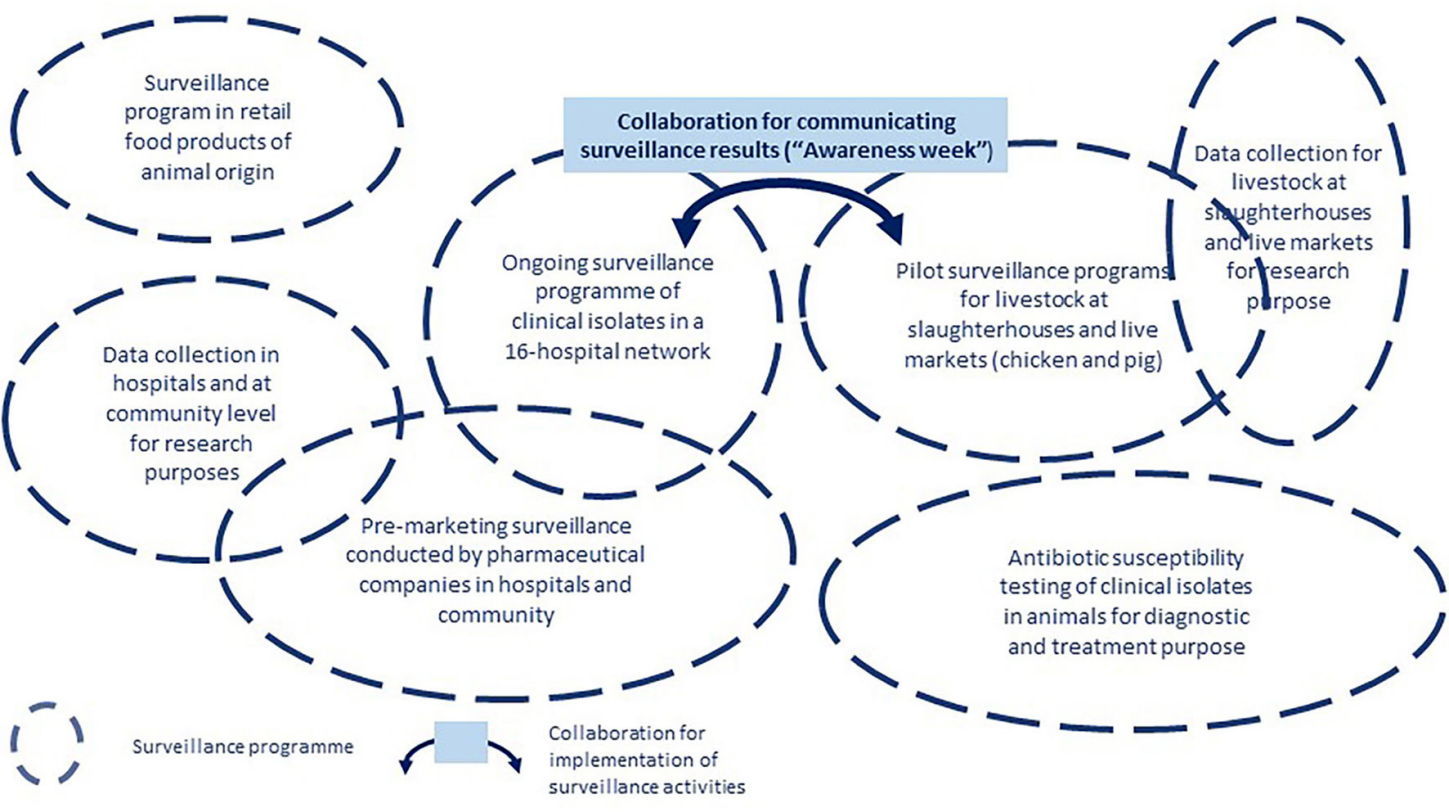
System map of the surveillance of antimicrobial resistance in Vietnam.

The stakeholder diagram was then used to produce the system map (Figure 6). This figure underlined that certain surveillance programs were covering the same domains and yet did not collaborate. The only existing collaboration among different programs was the joint use of surveillance results from hospitals and in animals during the public awareness week, under the impulsion of international organizations.

##### Surveillance System SWOT Analysis Results

The system's greatest strength resides in the presence of all the necessary structural elements at the surveillance program level to enable a functional multi-sectoral surveillance system (designated coordination units, functional laboratory network, etc.). The system also benefits from a strong political will, on behalf of national authorities and intergovernmental organizations, to combat AMR. Additionally, Vietnam has a culture and strong inter-institutional collaborative experience in the control of zoonotic diseases (rabies, avian influenza in particular) that can serve as a framework for the governance of the multi-sectoral AMR surveillance system. The surveillance programs show shortcomings in terms of governance (weak involvement of local authorities and insufficient resources) and operations (poor quality and unrepresentative data, too lengthy a reception time for laboratory results). At the system level, participants highlighted weaknesses in governance (steering, coordination, and scientific and technical support). The system also faces a number of challenges: the large number of stakeholders to be coordinated, the diverse format of data collected, the absence of government funding, the lack of involvement of certain governmental organizations and the lack of effective dissemination of surveillance results to decision-makers.

#### Desired Multi-Sectoral Surveillance System

**Figure 7:**
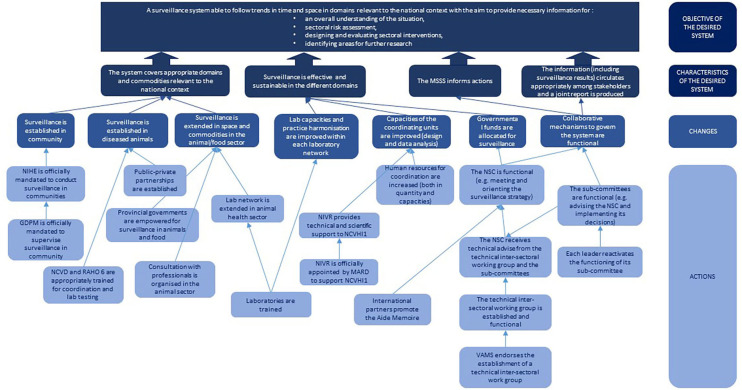
The innovation pathway constructed by participants to the participatory process in Vietnam. GDPM, General department of preventive medicine; MARD Ministry of agriculture and rural development; NCVD, National center for veterinary diagnostic; NCVHI1, National center of veterinary hygiene 1; NIHE, National institute of hygiene and epidemiology; NIVR, National institute of veterinary research; NSC, National steering committee; RAHO 6, Regional animal health office 6; VAMS, Vietnam administration of medical services.

The participants agreed that the priority was to produce relevant information within each sector and for each category of stakeholders in order to properly inform their decision and evaluate the effectiveness of the management measures implemented. Therefore, the participants defined the ideal surveillance system as a system capable of monitoring trends over time and space in all relevant domains, in order to improve general knowledge, inform sectoral risk assessment studies (including the correlation between use and resistance), support the development and evaluation of interventions in each sector and identify research needs. For such a system to be functional and sustainable, four conditions were identified: the system had to cover all relevant domains, surveillance had to be effective and sustainable in all domains, surveillance results had to be used to inform decision making, the different institutions in charge of coordinating surveillance had to share results and any other relevant information (Figure 7).

#### Necessary Changes to Achieve the Desired Surveillance System

Based on all the information produced during the previous steps, participants proposed different changes, which can be classified into three categories. The first was related to strengthening the governance of the multi-sectoral system and included: the existence of functional national subcommittees to steer and coordinate the system, the establishment of an inter-sectoral working group to provide scientific and technical support to governance mechanisms, the empowerment of local authorities in the animal surveillance network, the strengthening of coordination between authorities in charge of surveillance in food-producing animals and retail food and the establishment of public-private partnerships for the surveillance of clinical isolates. The second category consisted of strengthening technical and organizational capacities in the different existing surveillance programs. The third category was related to an increased coverage of the national surveillance system, through the implementation of surveillance activities of animal clinical isolates at community level, and the extension of the surveillance in food-producing animals to other commodities and geographical regions (Figure 7).

The group consisting of animal and food sector professionals worked on identifying actions to improve the capacity of the animal surveillance network, including analytical capabilities, and the inter-sectoral coordination of the national surveillance system. The group constituted of human health professionals worked mainly on defining actions to improve the inter-sectoral mechanisms for the steering, coordination, and scientific and technical support of the national system.

Figure 7 shows the causal links between actions, changes and characteristics of the desired One Health surveillance system to shape the innovation pathway toward the system objective.

In both groups, surveillance of AMR in ecosystems was mentioned and discussed. Both considered it was not a priority, arguing that ecosystems were contaminated by other compartments, either directly through resistant bacteria or indirectly through the release of antibiotic residues, imposing a selection pressure on bacteria present in the environment.

### Surveillance of *Salmonella* in France

In France, the participatory process was implemented during three half-day workshops, between April and October 2019. Participants were those present at the meetings of the technical group dedicated to *Salmonella* surveillance but varied over the course of the workshops as shown in Table 3. Because of this variation in the audience and of the long period between workshops, the restitution phase of the results previously produced was crucial at the beginning of the second and third workshop. This allowed newcomers to share their knowledge and view so they can be integrated into the co-constructed representations. Other participants used this opportunity to reflect again on the representations in the light of knowledge gained from group work's activities that had taken place between the workshops.

#### Definition of the Problem

The analysis of participants' expectations regarding their participation in the work group led to the definition of a first concerted objective (Figure 2). This was refined during the series of workshops, as the reflection progressed. The final objective was to produce strategic recommendations to improve the collection of data and the circulation of useful information in order to improve the management of the risk related to *Salmonella*. The problem was therefore 2-fold: on the one hand, the improvement of surveillance capacities by strengthening existing surveillance programs or by increasing surveillance coverage, and on the other hand, the improved circulation of information among all the stakeholders involved in *Salmonella* risk management, whether or not they are part of a surveillance program.

#### Representation of the Current Surveillance System

##### Stakeholder Diagram and System Map

**Figure 8:**
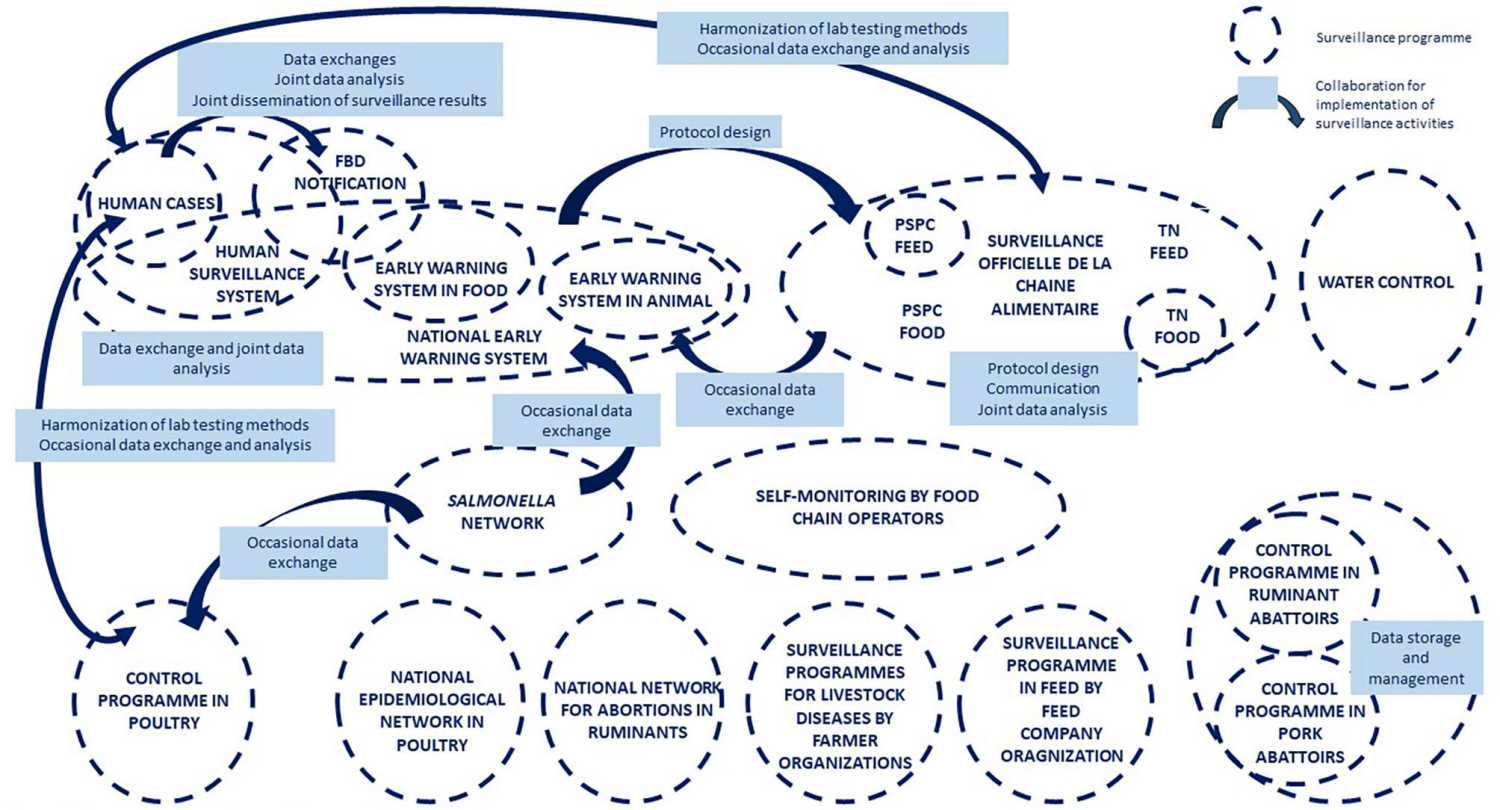
The System map of the surveillance of *Salmonella* in France.

The revision of the stakeholder diagram proposed by the research team led to the representation of a system that involved 41 different stakeholders operating in 18 surveillance programs (Figure 8). These stakeholders belong to the public (*n* = 28) and/or private (*n* = 19) sector, with seven working in both the private and public sectors. They fall into six professional categories: competent authorities (*n* = 14), private operators and professional organizations (*n* = 11), technical or research institutes (*n* = 8), testing laboratories (*n* = 7) or civil society (*n* = 1). They work in the sector of food production (*n* = 15), food safety (*n* = 14), animal health (*n* = 12), human health (*n* = 8), water production (*n* = 1), or ecosystem health (*n* = 1). The majority of programs (14/18) are sector specific, while others may cover two to four sectors. In total, eight programs cover human food, seven cover animal feed, six cover animal health, two cover human health, and two cover the environment. For the majority of these programs (12/18), coordination is ensured by public authorities. Twelve of them are of a mandatory regulatory nature, while the others rely solely on voluntary action. With the exception of water surveillance and a few isolates from wildlife collected through a laboratory network named “Réseau *Salmonella*,” there is very little surveillance activity concerning the natural environment.

The system map highlights the existence of a large number of collaborations for the governance and implementation of surveillance activities (Figure 8). However, the connections are not homogeneous within the system. While some programs appear to be isolated, others are highly inter-connected, creating sub-systems within the national system.

##### Surveillance System SWOT Analysis

According to participants, the major strengths of the current system are the regulatory obligations to report *Salmonella* detections, the strong mobilization of professionals to participate *Salmonella* risk mitigation, the existence of initiatives and mechanisms to allow for data mutualization and exchange, the existence of functional sectoral surveillance programs, and finally the participation in the surveillance effort of all professions and disciplines necessary for the implementation of an integrated approach. However, a poor articulation between existing surveillance programs and an insufficient circulation of information between stakeholders were highlighted. This was ascribed to the absence of collaborative mechanisms for the governance of the national system, which has a negative impact on the quality of the mitigation measures implemented. Surveillance requirements were considered uneven across production sectors (e.g., higher in the poultry sector) and insufficient in the natural environment to gain a good understanding of the transmission of the bacteria. The reconciliation of data from different sources is hindered by technical issues, such as disparity in format, the absence of a centralized system, and the non-systematic characterization of detected isolates. Participants identified a number of opportunities to be seized, such as the existence of functional surveillance programs in certain production sectors (e.g., poultry) that could serve as a model for other sectors, or the current national dynamics around the development of multi-stakeholder surveillance platforms (in animal health and food safety). In addition, it was stressed that substantial human, animal, and food strain characterization data were already available and could be easily compared and, in the future, the comparison between data should be facilitated by the development of new techniques such as high throughput sequencing. On the other hand, a certain number of challenges must be met to achieve the desired surveillance system: lack of resources, inappropriate communication in the event of a *Salmonella*-related health crisis, data ownership, mistrust, and fear of economic or administrative sanctions, which can represent a major obstacle to stakeholder involvement in information sharing and the need to change the attitude toward *Salmonella* risk (zero risk is not technically and economically sustainable for the sectors).

#### Desired Multi-Sectoral Surveillance System

**Figure 9:**
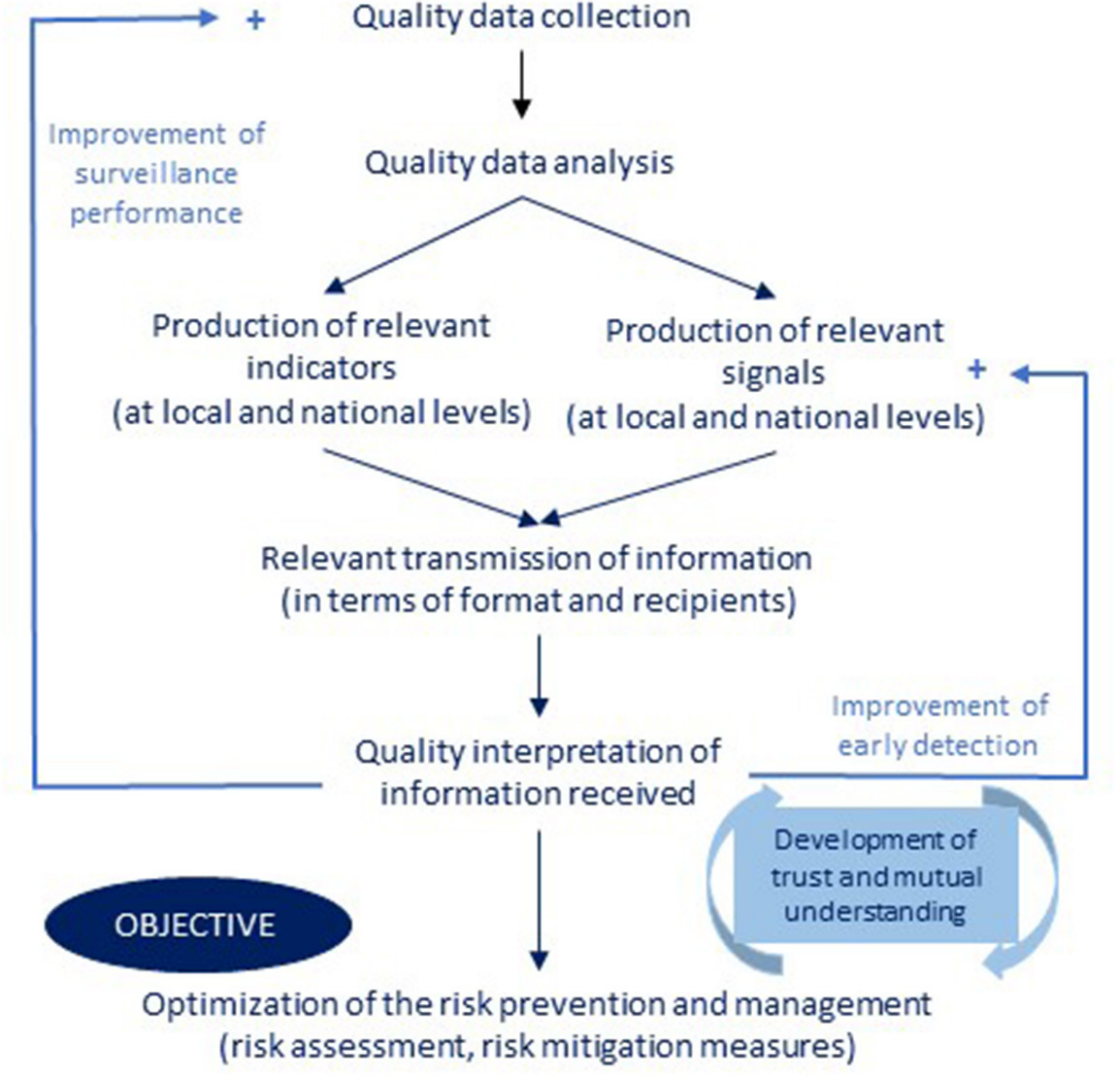
The representation of the desired surveillance system for *Salmonella* in France.

For workshop participants, the desired surveillance system (Figure 9) should be able to produce quality information, communicated to the right people in a timely manner, to achieve appropriate management and prevention measures. This involves the collection and analysis of high-quality data to produce indicators and signals that can be shared with risk assessors and managers (operators, authority, risk assessment agency). The implementation of appropriate measures to manage and prevent risk depends on the ability of information users to correctly interpret these indicators and signals. Sharing information between stakeholders should strengthen mutual trust between them, which, through positive feedback, should contribute to improving the flow of information.

#### Changes Required to Achieve the Desired Surveillance System

**Figure 10:**
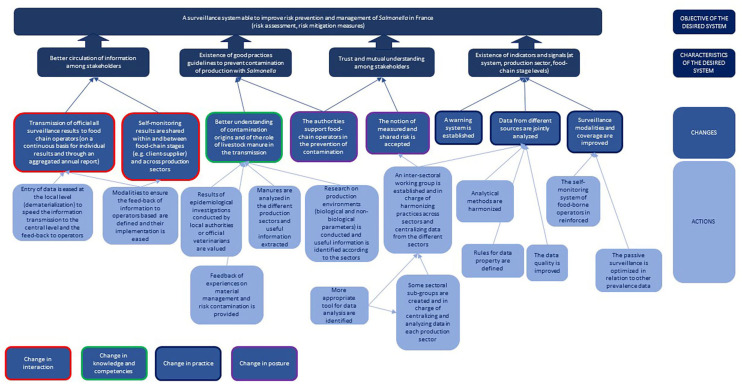
The innovation pathway constructed by the participants to the participatory process in France.

Participants identified major changes in the different pre-defined categories (Figure 10). In terms of practice, they identified the need to improve the modalities and coverage of passive surveillance, to increase the number of tests done by the food chain operators, and to set up an event-based monitoring system. Concerning knowledge, it appeared necessary to better understand the sources of contamination and the role of the discharge of farm effluents in the transmission of the bacteria. With regard to interactions, the results of official tests (positive and negative) should be transmitted to operators. In the same way, operators should share their results with operators working at the same stage of the food chain but also in other food-chain stages (e.g., between suppliers and clients). Finally, in terms of posture, operators should be better supported by the authorities. The adoption of a notion of measured and shared risk should replace the notion of zero risk.

The changes and the actions identified for their operationalization were combined with the representation of the desired surveillance system to draw the innovation pathway reflecting participant views (Figure 10).

## Discussion

### Participatory Process: Benefits, Caveats, and Facilitation

The participatory process developed and applied to the two case studies demonstrated its ability to engage multiple stakeholders with very different expectations and contrasting technical and social resources. This engagement allowed them to define a shared vision of the desired system and to negotiate the construction of an associated innovation pathway in which each of them could reasonably take part. Although the framework is developed here for the purpose of One Health surveillance, it may be efficiently adapted to other complex systems that require consultation between actors in a context of high uncertainty. Actually, the tools and methods mobilized here have been first developed to support collective decision-making in situations of conflict over resources (18, 21, 25).

The case studies highlight the applicability of the approach at two distinct levels of participation. Indeed, in the case of Vietnam, the problem was defined by the research team and the participants were invited by the latter to address it. In the case of France, the process was implemented at the request of the work group, which had identified the need to benefit from external support to better define and address the issue. Hence, referring to Pretty's ladder of participation as reviewed by Cornwall (28), the Vietnamese case started as a functional participation and evolved toward an interactive level by leading actors to take a part in own goals definition. In France, the process was triggered by the actors themselves, hence corresponding to self-mobilization. This different level translated into the process could be expressed as a “gradual empowerment” in Vietnam and as an “external coaching” in France.

While the framework is a well-defined structure in four main steps (Figure 1), the way they are approached, articulated and facilitated may differ, depending on the context and the information gathered during the process (Table 1). As with any participatory approach, the framework is flexible and iterative to adapt to the context of implementation and to accommodate the knowledge shared by the participants, as well as their position and reaction to the process (29, 30). In doing so, the change process toward One Health surveillance is adapted to the system's degree of maturity, in terms of method, goals, and actions. Through its inductive approach, starting by the consolidation of knowledge on the current system, the proposed method automatically adapts to the systems' maturity, strengths, peculiarities, and needs.

The success of such a process depends on the participants involved and the facilitation quality. As in any participatory approach, the representativeness and legitimacy of participants and their adherence to the process remain important issues, as these will impact the quality and relevance of the results (31). Special attention must therefore be paid to the selection of participants and to all the factors that can influence their commitment to the process (time and place of workshops, legitimacy of the organizing institution to initiate such an approach, etc.). In our two case studies, not all categories of stakeholders were represented throughout the process and this must be considered when referring to the workshop outputs for further activities (see section Role of the Process in Enabling Changes Toward One Health Surveillance). In order to overcome this issue, alternative solutions could have been implemented, such as individual consultation of the missing persons and *a posteriori* integration of their knowledge and point of view during the next workshop, after validation by all participants. Then, facilitation quality lies in its ability to accompany the production of knowledge and collective solutions (23). As experienced through the two case studies, the facilitation team may gain from involving three individuals with different postures: a “champion” who is recognized by the participants as legitimate to lead the process (working group coordinator, recognized teacher-researcher), a “naïve” individual who is in a comfortable position to invite participants to clarify and explain their discourse, and an “expert” who formulates relevant probing and follow-up questions. The role of the facilitator is also crucial in ensuring that each participant has a voice in the process. He/she must be able to manage conflicts and power games that may exist between participants, as well as the diversity of temperaments that may co-exist and be an obstacle to the collective process (32). As the proposed approach is adaptive and iterative, facilitators must be flexible in their methodology and be able to readjust their position and the way they carry out the different steps of the process as it unfolds.

### Role of the Process in Decreasing Uncertainty Related to One Health Surveillance

The two case studies highlighted the complexity for participants to envision their expectations regarding stronger collaboration and to define required changes for this collaboration to happen. The complexity, as a system characteristic, arises from two main features of the situation: the diversity and number of stakeholders and of their interactions, and the overall uncertainty around the objects under scrutiny. A major uncertainty does indeed prevail around stakeholders' expectations regarding the integration of knowledge and information in a One Health approach. Moreover, it proves difficult for them to anticipate the costs and benefits associated with their involvement in such an evolution of the system. One role of the process is to enable a joint and gradual mastering of the complexity of interactions through shared representations and mutual understanding, and to reduce uncertainty around the desired evolution of the system, by building a group definition of the required integration and relevant operationalization of One Health principles.

The framework is a process of translation and explanation in which participants are encouraged to accurately describe their knowledge of the different elements of the system and to mutually share this information. They have to explain who, in their opinion, are the key stakeholders, their role in the system, the interactions between them, the resources they exchange, the workflow and information flow, the power games at play, the institutional and operational issues and the problems they face. This leads to the formalization of a common language, then mobilized to produce a new shared representation of the whole system. During this process of deconstruction/reconstruction, participants systematically bring knowledge that will decrease the level of uncertainty regarding the expected outputs of the new system, the role of each stakeholder in it, etc.

The process also reveals challenges that stakeholders will face if they engage in the One Health surveillance system, so they can be discussed and anticipated. Meanwhile, the resources to be allocated to overcome these problems can be identified. Elements that would make the One Health system an attractive improvement are highlighted, leading to an understanding of the benefits and costs linked to the changes in practice (15). Finally, discussions make it possible to assess whether integration is feasible, while respecting or maintaining the diversity of co-existing purposes (33).

### Influence of the Surveillance System's Maturity on the Process Outputs

These two case studies tackle surveillance systems with contrasting degrees of maturity, as they are under development in Vietnam and already well-established in France.

Despite the differing maturities of their systems, participants in both cases emphasized that the performance of a One Health surveillance system depends essentially on the quality of each of the sectoral programs that are to integrate. Hence, in Vietnam, despite the pressing plea of international organizations in favor of a fully integrated AMR surveillance (3), the participatory process allowed participants to affirm their own positioning centered on more basic needs within each of the One Health components. Thus, they considered the strengthening of surveillance capacities in existing programs as a priority in the mid-term, before considering any data integration. In France, the quality of the data produced by the 18 existing programs was also identified as an essential prerequisite to achieve the objective of the desired One Health surveillance system. Interestingly, integration itself was then considered under the lens of an increase of information utility.

The question of information utility was tackled in the French case study from its user's standpoint, an aspect that was absent from workshops in Vietnam. This sharp contrast was directly linked to the system's maturity. The French system's greater stabilization, in terms of information production, allowed stakeholders to better focus on its use and impact. This user-based vision of health surveillance value and required improvement appears to be a quite recent concern, with methodologies that remain to be elaborated (13). Hence, participants proved able to develop original insights on the operationalization of the One Health concept in surveillance. Beyond collaboration between surveillance programs, the participatory process re-asserts the surveillance system's societal mission, acknowledging the diversity of stakeholders involved in risk prevention and management.

### Role of the Process in Enabling Changes Toward One Health Surveillance

The proposed method is an inductive and socio-constructivist action-research tool. Its objective is to capture the diversity of participants' knowledge about the system, stakeholders' practices, posture and capacities, and the interactions between them. On this basis, new knowledge emerges by combining and aggregating these sources of information, leading to the construction of shared visual representations (stakeholder diagram, system map, innovation pathway, etc.). These representations constitute together a conceptual framework to which participants can reasonably adhere. It is therefore not so much the conceptual framework in itself as its collective development that is expected to enable change (23). Indeed, during this development, participants are engaged in a social learning process that leads to a shared understanding of the situation and of the desired future (34–36). They have to listen to each other, make the effort to translate their ideas so that they are intelligible to others, and change their understanding and view of the current and desired system in order to integrate the information expressed by the others. This social learning process leading to the co-constructed and negotiated conceptual framework is expected to be conducive to the emergence of the collective action toward a One Health surveillance system (37).

As for other processes relying on knowledge co-production, the proposed framework has envisioned impacts in terms of collective actions. However, it does not have the capacity to measure them (33, 38). Indeed, it does not ensure that the innovation pathway constructed is the most appropriate one, that the actions identified are the most relevant or that the changes will actually take place. It represents a basis for later steps, which will ascertain or correct the intended plan, also through the inclusion of additional stakeholders who did not take part to the co-construction of the innovation pathway (e.g., local authorities). After revision, consolidation and prioritization of identified actions, the innovation pathway can be used as a working basis to develop operational recommendations and an action plan for the implementation of the desired surveillance system (21). Simulation exercises in the form of role-plays or board games can also be organized to test the proposed modalities, identify gaps and redefine them if necessary (36). Subsequently, an evaluation of the collaboration should also be envisaged, to check for the validity of identified pathways, their degree of realization and their re-orientation where needed. Obviously, these later activities would all gain from adopting the same participatory approach and could be included in the current framework, creating an additional step for the monitoring and evaluation of the system's development.

In Vietnam, no concrete action was taken following these collective workshops, even though the participants recognized that they had gained knowledge and mutual understanding and forged strong interpersonal relationships that would be beneficial for future collaboration. In France, following this work, a new workshop was organized to propose concrete and operational actions based on the outputs of the participatory process. A permanent work group dedicated to *Salmonella* surveillance was then established with the mission of coordinating the operationalization of these actions. This work group is transversal to the French epidemiological surveillance platforms for animal diseases and for the food chain, which includes representatives of the human health sector. This difference in impact between the two case studies is likely to be related to the degree of maturity of the system.

## Conclusion

The participatory process described here produces a conceptual framework that can be mobilized to generate collective action. As in transdisciplinary processes, the outcomes of the framework are not predetermined (33). This makes necessary to adapt the means of its implementation to the context and to remain flexible throughout the whole course of the process. Its objective is not to go as far as developing a detailed action plan for change implementation, but to create an environment conducive to discussion and to generate technical elements that stakeholders can then use to plan their future actions. The consultation and negotiation process initiated through the participatory workshops lays the foundation for a new partnership working toward a more integrated approach to surveillance, in which road maps can be produced and collaborative actions planned. A major challenge of this type of approach is to identify the exact nature of their impacts in terms of collective actions, leadership and decision-making, and to develop robust methods to measure them.

## Data Availability Statement

The datasets presented in this article are not readily available because data cannot be shared before previous authorization from participants to the collective workshops. Requests to access the datasets should be directed to marion.bordier@cirad.fr.

## Ethics Statement

The studies involving human participants were reviewed and approved by the Ethical Review Board for Biomedical Research of the Hanoi University of Public Health in Vietnam and the Ethical Committee for research of Paris-Saclay University in France. The patients/participants provided their written informed consent to participate in this study.

## Author Contributions

MB and AB designed and coordinated the study, analyzed the results, and drafted the manuscript. FG initiated the study, participated in its design, contributed to the analysis, interpretation of results, and to the finalization of the manuscript. PP-D and RL participated to the implementation of the study, the analysis of the results, and in finalizing the manuscript. NA-M participated in the discussion of the results and in finalizing the manuscript. All authors read and approved the final manuscript.

## Conflict of Interest

The authors declare that the research was conducted in the absence of any commercial or financial relationships that could be construed as a potential conflict of interest.

## Footnotes

^1^https://www.commod.org/en/qui-sommes-nous/association.
